# Always against the Woman

**Published:** 1878-10

**Authors:** Ausburn Towner


### ALWAYS AGAINST THE WOMAN.[1]

### One of Two Wrongs that Sadly Needs Righting.

BY AUSBURN TOWNER.

PART I.

There is no doubt about it. The world grows better every day. There is an improvement in all ways of human endeavor. The deepest rooted prejudices and notions, even if they are imbedded in rocks, if they are at all awry from the general good sense prevailing, must be torn up and thrown away to perish. Some one, seeing the wrong, will assuredly point it out and keep digging away until it disappears. The Law affords, at the present time, the greatest number of examples of a clinging to ancient notions, that the world generally has outgrown; notions founded upon an entirely different state of society and a different order of things. This is no more than natural, perhaps, for the Law, with a capital L, is considerable of an old woman, that sits quietly observing events and very seldom daring to tread in any paths except those that are well-worn. She is not much of an explorer. She has a pleasing and self-satisfying fiction, that she assiduously nurses among a million or more of other fictions, that she is the summit of wisdom, reason,[2] experience, justice and learning, and when confronted with something that is clearly unreasonable and unjust, as she frequently is, she calmly folds her hands and observes with a dignity that forbids reply:

"It is the Law and has a multitude of precedents to sustain it."[3]

There are a great many, nevertheless, who fail to comprehend fully why it happens, that because four or five hundred years ago, or longer, some Justice, in a little Island just the other side of the Irish Channel, decided in a dispute between two neighbors, that a certain course of conduct was right and should prevail, that we living in entirely different circumstances, should be bound by it. Yet that is a good description of what is known as the Common Law.

That this failure of comprehension meets with many active sympathizers is seen in the fact, that numerous enactments have been placed on our Statute Books, directly contravening notions that have been prescribed since "the time when the memory of man runneth not to the contrary."[4] Public opinion has pounded upon them so continuously and successfully that they had to go under, and public opinion should keep on until all of these wrongs are set right. A conspicuous example of the healthy change is found in the laws regarding the rights of married women. Every woman should know of and be thankful for it."[5]

One of the still-existent wrongs of which I would speak, is nearly allied to this one, so happily remedied, having also to do with the social relations, and appealing the stronger to our sympathies as it concerns those more helplesss than were women under the Common Law. It regards the custody of young children, when a question arises, as there frequently does concerning it.

Observe the law as it stands at present! It gives to the father of the child its charge, after it arrives at a certain age, no matter what may be the circumstances of the case. Is not this vitally wrong? Is it not unnatural, unreasonable and unjust?

A brief glance at these points, none of which requires much amplification to make it clear, will be conclusive. A mother is the natural guardian of a child, either boy or girl, until it arrives at an age when it can provide for itself, and this reaches to a period of sixteen or eighteen years. This makes itself manifest in all animated nature. Man

---

1. Genesis III—12. Proverbs XXXI—31. Of course every one has a Bible.

2. Reason is the life of the Law; nay, the common Law itself is nothing else but reason .........The Law, which is the perfection of reason.— *Coke Institute, Book* 1. *Fol.* 976.

3. If, in one way or another, there comes a change, she can still retain her position, with perhaps a reverse action of her thumbs, and with undisturbed equanimity can make reply in nearly identical terms: "It is the Law.'

4. Blackstone's trite shibboleth.

5. Six years ago, in one of our eastern cities, a depraved and useless husband abandoned his wife and his two bright boys, leaving them utterly destitute. She was a self-reliant woman, and obtaining assistance from some kindly disposed persons, opened a grocery, where she sold small quantities of everything. The neighbors, knowing her situation, gave her their custom and she prospered so well, that in a short time she had a well-filled and well-appointed store that furnished her with a comfortable living, and also permitted her to accumulate something for the benefit of her children. One day last summer, the husband whom she had not seen nor heard of for six years, walked into the place with a constable, pulled off his coat, stepped behind the counter, and announced himself the proprietor of the concern! What was simply unlimited cheek in these days, thank Heaven! under the Common Law that has a million of precedents to sustain it, would have been only the assumption of a legal right. The miserable wretch could have claimed and had his claim allowed for, the result of his wife's six years' hard and patient labor, and under the same rule, could have turned her out of doors, or if she objected in what he might consider to be an improper manner, he could have beaten her with a "stick as thick as his thumb!" But that is changed now, and a warm feeling of gratitude comes over us as we learn the result of his attempt. The wife procured a warrant for the arrest of the interloper, who had, under any law, about as much right in the store, as the English have in Cyprus, on the charge of being a vagrant, without visible means of support, and he was sent to jail for ninety days. Nevertheless, the feminine nature was well demonstrated in the expressions of the wife. She pitied the man sincerely, and said that if he would only behave himself he should have a home with her and be provided for. It came out also, that some years before, he turned her out of doors in a midwinter night, yet she had no bitter words to utter against him.

Is this not a change for the better?

forms no exception to the rule, but is, in fact, one of its strongest illustrations, for a mother's oversight of her children never slackens while she lives. In other species it passes away as soon as the offspring no longer stands in need of her personal attention. We have all had mothers, and each one can attest the truth of this. Be you ever so old, and you are still happily blessed with the daily sight and conversation of that ever watchful being, who, in the midst of pain and anguish gave you life, and there never is a time when you cannot go and creep under her wing and feel that you can always find comfort and sympathy there, but who ever saw a chicken flying for protection to the lordly cock strutting about a barnyard, whose every feather seemed to say, "Stand aside, sir!"

I do not say, I could not say that fathers do not love their children. That would be very far from the truth. There are multitudes of good fathers who toil in season and out of season for their offspring, wearing themselves out, in frequent instances, in the struggle to place them in a position where they can commence the world with fair, if not brilliant prospects; making sacrifices daily that no one shall ever know until the final account comes to be settled. I do not allude to any of these, nor does this paper, in any of its parts, have the remotest reference to them. The strength of the Law is sin, and this Law about which I write, like every other law, crosses never the path of the good. Laws are made for the evil and it is because of the evil that there is law.[6]

I would like to interject something here, also, that is not entirely foreign to the general theme. There has always in common life and literature, been a great deal of what might be called "Mother-talk." The astonishing love, surpassing all other love that a mother has for her child. It will be recollected that this reached such a nauseating pitch during the war, when the songs of "Mother take me home to die," Mother this, and Mother that, that this really most sacred sentiment and feeling stood in danger of becoming a subject for reviling and contempt. Fortunately it escaped, but not without a severe shaking. But it has always prevailed to such an extent that you would hardly think that there was such a being as a Father. Now that which has such a universal currency amongst the whole human race, must have something to bottom it. Granted that fathers do love their children, the mother's love is so immeasurably greater and deeper that it so overshadows the other as almost to make it appear that it had no existence. And that brings us back.

A mother's feeling for her child is natural, positive, her own, something that the metaphysicians would call subjective, that is, springing from her own inner consciousness, going out from her and not coming from other sources to her. A father's feelings are reflected, coming more from habit, objective, brought to him, not given from him. The child is a part of its mother, and her love for it begins before it is born. It is a long time after its birth before a father, any father, can truthfully be said to love his child. This can be affirmed with safety, and, in my observation, I have found no father who would gainsay it. It is all cant, rubbish and affectation, this bringing a baby, shortly after its birth, to its father, as though they were bringing to him something in which his whole soul was wrapped. His thought is for anything just then, except the baby. His whole heart is for the dear creature up-stairs, sick and weary, who has been passing through that to which the valley of the Shadow of Death is a mere trifle. A baby, his own or the property of any one else, is absolutely disgusting to a man. Love it! Why he can love an angleworm or a lump of unbaked dough with as good a stomach!

In time though, when the child begins to make it manifest that it belongs to the order of intelligent beings instead of the mollusks, and the mother once more gets rosy-cheeked and bright-eyed, the father looks upon his babe. He sees how much the mother loves it, and he begins to love it for her sake.[7] It is hers, a part of her, and her love for it is reflected upon him.[8] Pres-

6. That is good orthodoxy too, for which consult your Testament and Catechism.

7. Is not the idea, that the love of the father for his children comes through the mother, that I have endeavored to set forth, borne out in many ways? The thought and truth of centuries, is sometimes boiled down into one sentence, and what does the ancient couplet

"A mother 's a mother as long as her life,
But a father 's a father till he gets a new wife,"

mean if it does not mean what I have been putting in just as homely prose? It may be a source of much unhappiness, so is a bile, but it is natural, nevertheless.

8. It is an ancient and time-worn legend, but apt, just here. A great King fell into deep trouble, and his astrologers and wise men made it known unto him that by giving up to be burned to death that being that he loved the most, his troubles would be dissipated. Now the King loved his wife above all other persons in the world, and when he heard the proclamation of the wise men, he grieved that it should be so and studied how he could answer the demand and yet save her. There had been born to them a son who had reached to the age of eight years, and on him were the hopes of the King and his people centered. And the King, after much delay and grievous wrestling with his spirit, and having had repeated communications from the gods reiterating the demand made by his wise men, determined that his son should answer for him as the being he loved the most on the earth. Then great preparations were made for the sacrifice, and at the appointed time, the King with his train and the Queen with her women, assembled on the plain to witness it. The Priests chanting hymns to their gods led forth the youth to the pile prepared. But the ently he forgets how the love originated and learns to love the child for its own sake.

Take it further on. A father never enters so completely into the life of a child as a mother; never knows it so thoroughly, its disposition, failings or excellencies. He is, therefore, not so able to correct and warn against the one and strengthen and build up the other. He has another and a more enlarged field in the world. His supervision of his offspring, at the best, can be only of a general nature and at brief intervals. It cannot be constant and personal as a mother's. He can philosophize as to what is best to be done, and direct that it shall be done, but he cannot do it. This is not to be complained of, it is only natural, and so makes for our point. His children, while they are always close to the mother in every sense of the term, are always distant from him. Let this be made personal. Do you, gentle reader, know your father as well as you know your mother? If anything is greatly coveted from the paternal side, do you go direct to that august source and solicit it, or do you not, the rather, first discuss the subject with the one who you know beforehand, will sympathize with your desire, and will act as an ambassador that seldom fails of success? You can make but one answer to this. Why there are many men and women who never get acquainted with their father, giving the old saying, "It's a wise child that knows its own father," a much wider signification than the vulgar one usually applied to it. It is a very common thing to see even good fathers, who are social princes, lively in conversation and quick at repartee, who, in the presence of their children, put on a solemn, patriarchal air, that is positively forbidding. Did you ever know a mother of this character?

If these things are true that I have set forth, and it would be a difficult matter to refute any of them, is it not conclusive that a child should follow its mother and that any law making it otherwise is unnatural?

The law is unreasonable. It may have originated in reason, or what was thought to be reason at the time. We do not so regard it now. It began in those desperately forlorn times when a wife was considered—it is difficult to describe her position intelligibly. A cipher would perhaps briefly express it, and yet would not, for she was something and yet nothing. She had an independent existence only in the fact of her being alive. The old Norman law phrase, *femme covert*, perhaps describes her more accurately than other words. It has no exact synonym in English. She was a woman covered up, swallowed, snuffed out by her husband. If it arouses one's impatience to examine those barbarian, and in these respects, contemptible ages, how much more should it excite a deeper feeling than impatience to see certain rules of conduct called law, extant then, surviving in this enlightened day and generation.

The injustice of the law makes itself apparent in almost every day of the year. It comes oftentimes before the trouble in the family is made public or even reaches the ears of relatives. More than one wife chafing in the shackles that binds her to a brute, has been awed and crushed into submission, and bears her chains until they drag her into the grave, by the repeated threat: "I'll take your children away from you." And this contemptible law virtually pats him on the back and says: "Go ahead, old chap. Make it hot for her. I'm right behind you." And he knows that it has much more power and force than if he had said: "I'll stick a knife in your heart."

Then, if the unhappiness and wrong have a flood of light thrown upon them in the courts so that all may gaze at one phase of imperfect human nature displayed in its deformity, the custody of the children of the mismated couple, is seldom heard of, except that a sum of money is involved; the child, if a girl, is desired for some vile purpose, or the man exults in intensifying a misery of which, in nine cases out of ten, he has been the author. The Law permits him to succeed, whatever his reason. Always against the woman. If the wife has been the faithless one, it is set up that she is not a proper person for the care of the little ones, and they are, perhaps, justly taken away from her. But if the husband is the guilty one, no such allegation is made against him, yet the mother must lose her children all the same, if the father demands them. Is *that* just? The question carries its own answer.

You will not need to look long for examples il-

---

Queen, who, until that instant, had been ignorant of what was intended, seeing the danger of her son, uprose from her throne, ran with the speed of the wind to the altar and falling upon her face before the servants of the Gods, prayed that they should spare the boy and take her life instead. And the Priests hesitated. But the King seeing the beloved of his soul in this position, himself descended from his seat and caught her in bis arms, waving the Priests away with his hands. Then said one of the servants of the Gods:

"Oh, King, live forever! Thou seest thy son in the hands of the executioner and yet art unmoved, but when thy wife approaches, lo! thou art stirred even as the hill-top in a strong wind. Take thy son. It is not he whom thou lovest best of all created things. Thy acts have given the lie to thy words and the wrath of the gods is unappeased.

It is altogether bad to draw an argument or illustration from the old slavery days, yet in those times, the child always went by law with the mother. That is, if a child was born to a slave-woman, her owner was its owner. But then laws, until very recent times, have always been against the woman unless the alleged reason for their existence is in the man's interest.

lustrating the truth of these propositions. Almost any newspaper published in any large city, will furnish plenty of them, and frequently you will be able to observe instances that mark the glaring injustice of the law with striking force.

Five years ago a childless couple adopted from a children's home a girl babe a few months of age. She was beautiful in feature and form, but in delicate health. Her mother consented to the adoption, sorrowing to part with what she was unable to care for properly, but happy that the future of the child would become so bright. The child was nursed into robust health, fulfilling in all other respects her early promises. When she was almost six years of age, a man, rather a wretch, appeared and claimed her as his daughter. The adopted parents had never so much as heard of him before. He was a drunkard and had deserted the mother of the child before it was born, leaving her in such want that she was obliged to go to a hospital. His just right to the little girl was so infinitesimal that there is no formula to express it. Nevertheless he claimed her. His claim was very properly refused. Then he went to a lawyer and obtained a writ of *habeas corpus*, upon which all of the parties appeared before a Justice of the Supreme Court. The man, in some way or other, satisfied the Justice that he was the little girl's father, and the Justice, under the unrighteous law could do nothing else than accord to him her custody. It seems hardly necessary to add, yet it more completely rounds off the story, that the grief of the child and the foster parents, especially of the foster mother, was extreme, for they had all become greatly endeared to each other.

Will some one be kind enough to inform me where the justice of such a decision comes in? Remanding a child to the care of an utter stranger who, before she was born, had voluntarily abandoned her, and taking her away from those who had loved and cared for her in her infancy, and who begged to be permitted still to love and care for her. Was it not competent for the Justice to inquire whether the suddenly appearing father was able to care for his newly assumed charge or what were his purposes in regard to her? Such inquiries did not enter at all into the question. And the further inquiry naturally arises, if the father had been offered $400 or $500 by the foster parents, would he not have been willing to give up a claim on one in whom an interest that had been lying dormant for five years suddenly awoke?

You never read of a case of this kind that you do not involuntarily pitch away the newspaper that relates it and feel that there is something wrong somewhere. There is, and it could be remedied so easily that it is a wonder our wise men who meet annually in Albany and spit tobacco juice on the floors of the Senate and Assembly chambers, have not yet applied it. It is not that the father should never have the custody of the child. That would be as ridiculous and unjust as the present way. Give the Justice some discretion in the matter. Let him inquire carefully into all the circumstances, and by all means, let the actions and words of the child itself have some weight. Oftentimes there is more care exercised in the examination of a question where a cow or a horse or a sheep properly belongs, than where a child does. Let there be some equity between the mother and the father. A father should be obliged, of course, to provide for his children, that is a natural duty that the courts should enforce, as they do, in cases where there has been no marriage,[9] but their custody should be in the sound discretion of the Justice, founded upon the testimony presented as to where the child or children would be the best off. If it were all happily changed, the details of cases that stir up one's soul in an uncomfortable manner, and that have worked more unhappiness and misery than any other one piece of inherited injustice, would no longer disgrace and disfigure the records of our courts.

9. Always against the woman, you see.

